# Help overcoming pain early, a brief person-centred intervention for adolescents with chronic pain in a school setting, may improve symptoms of insomnia

**DOI:** 10.3389/fpain.2023.1264355

**Published:** 2023-11-14

**Authors:** Ulrika Wallbing, Stefan Nilsson, Mari Lundberg, Helena Wigert, Mike K. Kemani

**Affiliations:** ^1^ Institute of Health and Care Sciences, Sahlgrenska Academy, University of Gothenburg, Gothenburg, Sweden; ^2^ Department of Neurobiology, Care Sciences and Society, Karolinska Institutet, Stockholm, Sweden; ^3^ University of Gothenburg Centre for Person-Centred Care (GPCC), Sahlgrenska Academy, University of Gothenburg, Gothenburg, Sweden; ^4^ Department of Pediatric Oncology, Queen Silvia Children's Hospital, Sahlgrenska University Hospital, Gothenburg, Sweden; ^5^ Department of Health Promoting Science, Sophiahemmet University, Stockholm, Sweden; ^6^ Division of Neonatology, Sahlgrenska University Hospital, Gothenburg, Sweden; ^7^ Medical Unit Medical Psychology, Theme Women's Health and Allied Health Professionals, Karolinska University Hospital, Stockholm, Sweden; ^8^ Department of Clinical Neuroscience, Karolinska Institutet, Stockholm, Sweden

**Keywords:** adolescents, chronic pain, insomnia, person-centred care, schoolnurses

## Abstract

**Introduction and aims:**

Chronic pain and symptoms of insomnia affect large numbers of adolescents and early interventions are prioritized. The aim of the current study was to evaluate potential secondary effects of the intervention, Help Overcoming Pain Early (HOPE), on symptoms of insomnia and self-rated health.

**Methods:**

The study included non-randomized aggregated data from the active and control conditions in a previously conducted randomized controlled trial evaluating the efficacy of HOPE, after the participants in the control condition also had received the intervention. Symptoms of insomnia were assessed with the Minimal Insomnia Symptom Scale and self-rated health was assessed with one item, at the start of the intervention, post intervention, and at a six-month follow-up. Baseline variables included age, gender, pain localization, pain impact, school absence and symptoms of depression (assessed with the Center for Epidemiological Studies Depression Scale for Children). Inferential analyzes were performed using Linear Mixed Models (LMM). Effect sizes were evaluated by calculating Cohen's *d*.

**Results:**

There were statistically significant improvements in symptoms of insomnia at the six-month follow-up, and statistically significant improvements in self-rated health at the end of the intervention and at the six-month follow-up. Effect sizes were small across outcomes and assessments.

**Discussion and conclusion:**

Results illustrated significant but small improvements in symptoms of insomnia and self-rated health in adolescents with chronic pain following the HOPE intervention. Although caution is needed when assessing the findings, results illustrate the potential utility of an accessible brief early intervention in a school context.

## 1. Introduction

Chronic pain and symptoms of insomnia affect large numbers of adolescents (1, 2). In addition to disturbed sleep, one study showed that approximately half of the adolescents with chronic pain also had impaired daily functioning (e.g., restricted ability to attend school, spend time with friends, participate in hobbies) as a consequence of their pain (1). As sleep is a critical aspect of health and well-being for adolescents with chronic pain (3), potentially, brief interventions provided in a school setting addressing these issues may comprise early effective and accessible treatment avenues for these adolescents.

Symptoms of insomnia include a non-satisfactory quantity or quality of sleep, persisting for a considerable time, including difficulties falling asleep, staying asleep, and/or early awakenings (4). Lack of sleep below the recommended eight to ten hours of sleep for adolescents may affect health and well-being (5) and negative consequences of prolonged periods of poor sleep include increased irritability, impaired executive functioning (6) and attention, lack of motivation, and frequently co-occurs with anxiety and depression (7). Results from a critical review summarizing the interrelationship of sleep and chronic pain in adolescents showed that more than 50% of participants reported sleep deficiencies, including difficulties falling asleep, maintaining sleep, feeling rested, and sleeping for an adequate duration (2).

Oftentimes the relationship between chronic pain and symptoms of insomnia is bidirectional (2, 8), meaning that pain may negatively influence sleep, but also, that poor sleep may negatively influence the experience of pain, indicating that effective pain management may need to address both pain and insomnia (2, 9). Notably, results from studies that have investigated the bidirectional effects of sleep and pain, suggest stronger support for sleep difficulties having a negative effect on chronic pain (2).

As adolescents spend a considerable amount of time in a school context, interventions provided in a school health care setting may be an effective way of reaching those in need. Also, in Scandinavia, school nurses are commonly the first line of health care, providing them with an opportunity to offer early health promoting interventions for adolescents (11), before the introduction of specific interventions provided in primary and tertiary care settings. However, school nurses express that they often lack adequate skills and conditions to provide interventions for adolescents experiencing chronic pain (10, 12). Taking the above into account, we see the development and evaluation of early interventions for adolescents affected by pain and related difficulties such as insomnia as a prioritized area. Thus, Help Overcoming Pain Early (HOPE) was developed, an intervention that could be provided by school nurses to support adolescents who seek support for their chronic pain (13). HOPE comprised four sessions provided in the schools by the school nurses. Based on a person-centred approach and a co-created health plan, the relationship between stress, pain and associated problems were discussed and strategies to manage pain, stress and sleep problems were explored (13). In a previously conducted randomized controlled trial (RCT), we evaluated the efficacy of HOPE, in comparison with a wait list condition, but found no effect on the primary outcome self-efficacy in daily activities (15). However, post-hoc analyses based on data from this study illustrated tentative effects of the intervention on the primary outcome for a subgroup of participants, adolescents in secondary school (15). At the end of the intervention in this previous study, adolescents in the waitlist condition were also provided the HOPE intervention. In the current study we aimed to evaluate the potential effects of HOPE on secondary outcomes, for all adolescents who received the intervention. These outcomes included symptoms of insomnia and self-rated-health. Self-rated-health was included given the importance of this factor as a determinant for illness and mortality, and the cause for concern as to the ability for adolescents to achieve their full health potential (16, 17). It is also worth noting that we did not conduct any follow-ups in our previous study (15), meaning that we may not have captured change that required more time to manifest. This aspect provided an additional rationale for conducting the current study, aiming to assess change over a more extended period of time.

## 2. Material and methods

### 2.1. Procedure

The current study comprises secondary analyses of data collected in conjunction with the RCT evaluating the efficacy of the HOPE intervention (15). In the present study we used data for all participants, i.e., including participants from the waitlist condition, after they had received the HOPE intervention. This means that we collapsed the previously randomized conditions, into one group of participants. Outcome measures were assessed at the start of the current study, post intervention and at follow-up six months after the end of the intervention. Participants were recruited and received the intervention between August 2016 and October 2018. Participants allocated to the waitlist condition received the HOPE intervention following post-assessment, and the final session of the intervention took place in May 2019.

### 2.2. Participants

The recruitment process, eligibility, as well as inclusion and exclusion criteria have been presented in detail in the article dealing with the findings from the previous study (15) and is thus briefly covered here. School nurses in 16 schools (private and public) were responsible for recruitment. The schools were situated in several geographical locations in 10 communities of varying sizes and socio-economic contexts. Eligible participants were students in secondary and upper secondary school, who had experienced pain for at least three months.

Inclusion criteria were: Pain, stress during two of the three last pain episodes of pain, rating at least 1 on the 6-point Verbal Rating Scale for Stress (VRSS), ranging from 0 to 5 (0 = no stress at all; 5 = worst stress) (18). Exclusion criteria were: Inability to speak and understand Swedish; concurrent participation in a conflicting study; and cognitive impairment. The study was conducted in accordance with the World Medical Association's Declaration of Helsinki (19) and received ethical approval from the Regional Ethics Review Board in Gothenburg (Registration number 172-16). The study was registered in Trials.gov Identifier as NCT02944786. Adolescents, as well as guardians of adolescents younger than 15 years of age, provided informed written consent to participate in the study.

### 2.3. The intervention—HOPE

The intervention has been described in more detail elsewhere (12, 15, 20, 21), but is briefly outlined below. HOPE was based on a collaborative approach involving end-users in the design process (22) and the content of the intervention was framed within a person-centred care framework. The school nurses underwent a one-day training program including lectures, written material and films on person-centred care, gender perspectives, neurophysiology and practical pain-, stress- and sleep-management strategies. The school nurses provided the intervention in the schools in four individual sessions, aiming to improve the adolescents' understanding of problems associated with chronic pain, such as symptoms of insomnia, and to help them finding personally adapted strategies to manage such problems. The intervention comprised two main activities. Firstly, it included the formulation of a person-centred health plan in which individual goals were set. Secondly, it included an educational component aiming to explain the relationship between stress, pain and associated problems based on a biopsychosocial perspective. This part also included personally adapted ways to manage pain, stress and sleep problems and included strategies for achieving structure, activity balance and recuperation, through for example physical activity and relaxation exercises. The school nurse's educational material regarding sleep included specific areas to be addressed in conversations with adolescents when discussing sleep problems. These included identifying factors that facilitate sleep such as adequate room temperature, a sufficiently quiet and darkened room and minimizing screen time and smartphone activity in conjunction to bedtime. In addition, information was provided on how napping, physical activity, food intake and certain medications affect sleep. Lastly strategies, such as relaxation techniques, were promoted to more effectively address evening and night time stress, worry and anxiety.

### 2.4. Background and outcome variables

Self-reported background variables included age, gender, pain intensity, pain localization, pain impact (i.e., how pain affected the adolescents), school absence and symptoms of depression. Pain intensity was assessed using a numeric rating scale (NRS) in which the adolescents rated the intensity of the most dominant pain on a scale from 0 (“No pain”) to 10 (“Worst pain possible”) (23). Pain impact was assessed with the question: “How does all the pain affect you?”. The question was rated on a 4-point scale including the options: 0 = not at all; 1 = a little; 2 = quite a lot; and 3 = a lot (13). To assess depression, we used the Center for Epidemiological Studies Depression Scale for Children (CES-DC) (24). The CES-DC consists of 20 items and item responses range from 0 to 3 and include the alternatives: (0) Not at all; (1) A little; (2) Some; and (3) A lot. Responses on individual items are summed together to determine a total score that can range from 0 to 60, and higher scores indicate a higher degree of depressive symptoms. The questionnaire has been validated in a number of studies, and results indicate adequate evidence of reliability, internal consistency and concurrent validity for adolescents (24–26). The outcome variables, symptoms of insomnia and self-rated health, were assessed using self-report questionnaires, and these are detailed further below.

#### 2.4.1. Insomnia

The Minimal Insomnia Symptom Scale (MISS) was used to assess symptoms of insomnia (27). MISS is a three-item questionnaire aiming to assess key symptoms of insomnia: (1) Problems falling asleep at night; (2) night awakenings; and (3) unrefreshing sleep (28). Each item has five response alternatives as to the severity of the symptoms: None; minor; moderate; severe; and very severe problems. Items are scored from 0 to 4 respectively. Hence, the total score ranges from 0 to 12 points, higher scores indicating more severe insomnia symptoms. Results from a previous study investigating the questionnaire's measurements properties in a sample of adolescents found general support that MISS had good fit to the Rasch model they used (29). Based on their analyses, these authors suggest a cut-off score of ≥6 for identifying insomnia in adolescents using MISS (29). This cut-off score was also suggested in another study including adults (27). Additionally, this study also showed that MISS was able to distinguish subjects with clinical insomnia according to ICD-10 research criteria and that test-retest reliability was found to be adequate, as shown by an ICC coefficient of 0.79 (27). In the current study we used the above suggested cut-off score, i.e., a total score on MISS ≥6, to identify insomnia among participants.

#### 2.4.2. Self-rated health

Health (Self-Rated Health: SRH) was evaluated using the single item: “How do you rate your general health?”. Possible responses range from 0 to 4 and include the alternatives: (0) Very poor; (1) poor; (2) neither good nor poor; (3) good; and (4) very good. Thus, higher scores indicate greater self-rated health. SRH is repeatedly used in research assessing overall subjective perception of health. A number of studies with adult participants have evaluated different aspects of validity of single-item SRH-measures, providing support for criterion validity by means of expected associations with BMI, physical exercise and frequency of drinking alcohol (30), and strong predictive validity in regard to mortality (31), health care utilization (32), and morbidity (33). For adolescents, SRH has been shown to be a relatively stable construct during adolescence, and corresponds as expected and consistently with a lack of general well-being, disability, healthcare attendance and health-compromising behavior (34).

### 2.5. Analytic procedure

Inferential analyses were performed using Linear Mixed Models (LMM). Under the assumption that data were missing for ignorable non-random reasons, Maximum Likelihood (ML) estimation was used to model parameters and standard errors across the three time points, based on all participants who provided at least one valid assessment for the dependent variables (i.e., intention-to-treat analysis). Assumptions of normality were tested by evaluating deviations from the normal distribution of the residuals in the model. Time was included as a factor (and not a covariate) to better manage the non-linearity of data across the assessment points. Both models controlled for the schools the participants attended, age, gender baseline symptoms of depression and the baseline levels of the specific outcome being analyzed (MISS and SRH). Furthermore, post-hoc tests (*F*-tests) were performed to evaluate the effect of the intervention based on pairwise comparisons of the estimated marginal means using Bonferroni correction. Within-group effect sizes were calculated according to Cohen (35), based on change-scores (pre- to post- and follow-up-assessment) and estimates and standard errors (SEs) from the mixed-model output. Effect-sizes (*d*) were categorized as small (*d *≈ 0.20 to <0.50), medium (*d* ≥ 0.50 to <0.80), and large (*d* ≥ 0.80). All analyses were performed using IBM SPSS for Mac version 26 (IBM Corp. Released 2019. Armonk, NY: IBM Corp). Restrictions apply to the availability of the dataset due to the European General Data Protection Regulation (GDPR).

## 3. Results

### 3.1. Baseline participant characteristics and questionnaire scores across assessment points

As regards missing data, 13.3% of the assessments with MISS were missing at post-assessment and 28.6% of the assessments were missing at the six-month follow-up. Regarding SRH, 10.2% of the assessments were missing at post-assessment and at the six-month follow-up 28.6% were missing. Of the 98 participants included in the current study (15), 92 (93.9%) participants completed pre-assessment on both MISS and SRH. Of those that completed the pre-assessment, 82 (89.1%) participants completed post-assessments on both outcomes, and at the six-month follow-up 65 (70.7%) participants completed assessments on both MISS and SRH. In the mixed model analyses, all participants with at least one assessment were included in the analyses (MISS, *n* = 93 and SRH, *n* = 94). In total 91 of the participants completed all four sessions. Of the included participants 76 (79.2%) were girls. The most common singular pain localizations were the head (57.7%), abdomen (24.7%) and back (7.2%). As regards symptoms of insomnia, 54.3% of the included participants had a MISS-score ≥ 6, used as a cut-off for insomnia in the current study. Please find additional background characteristics of the participants in Table 1. Observed average scores for the included outcome variables assessed over time are presented in Table 2.

**Table 1:** Baseline background characteristics for the participants in the current study.

Variables	Female	*n*	Male	*n*	All participants	*n*
	*n* (%)/ Mean (SD)		*n* (%)/ Mean (SD)		*n* (%)/ Mean (SD)	
Gender^a^	76 (78)		22 (22)		98 (100)	
Age	16.1 (1.52)	75	17.1 (1.27)	22	16.3 (1.52)	97
Pain intensity	7.4 (1.7)		6.8 (1.47)		7.3 (1.67)	98
Pain localization		75		22		97
Headache	44 (58.7)		12 (54.5)		56 (57.7)	
Abdominal pain	21 (28.0)		3 (13.6)		24 (24.7)	
Back pain	4 (5.3)		3 (13.6)		7 (7.2)	
Other	6 (8.0)		4 (18.2)		10 (10.3)	
How does pain affect you?^b^		76		21		97
Not at all	1 (1.3)		0		1	
Little	11 (14.5)		6 (28.6)		17 (17.5)	
Quite a lot	37 (48.7)		11 (52.4)		48 (49.5)	
A lot	26 (34.2)		4 (19.0)		30 (30.9)	
Has pain hindered you from attending classes?^b^	56 (74.7)	75	18 (85.7)	21	74 (77.1)	96
Symptoms of depression	28.5 (9.63)	74	28.1 (11.62)	22	28.4 (10.06)	96
Symptoms of insomnia (miss-score ≥ 6)	40 (54.1)	74	11 (55.0)	20	51 (54.3)	94

The participant numbers vary as different participants failed to provide information across the included variables.

^a^ In the current study we did not collect information on gender identities other than female or male.

^b^ The question was asked without specification of a certain timeframe.

**Table 2:** Means and standard deviations for symptoms of insomnia and self-rated health across assessments.

Assessment point	MISS	*n*	SRH	*n*
	Mean (SD)		Mean (SD)	
Pre-treatment	5.74 (2.80)	94	2.35 (0.77)	96
Post-treatment	5.22 (2.97)	85	2.66 (0.83)	88
6-month follow-up	4.77 (2.60)	70	2.70 (0.92)	70

Variables and questionnaires include: Symptoms of insomnia, MISS, minimal insomnia symptom scale and self-rated health; SRH, self-rated health.

### 3.2. Changes over time in symptoms of insomnia and self-rated health

**Figure 1:**
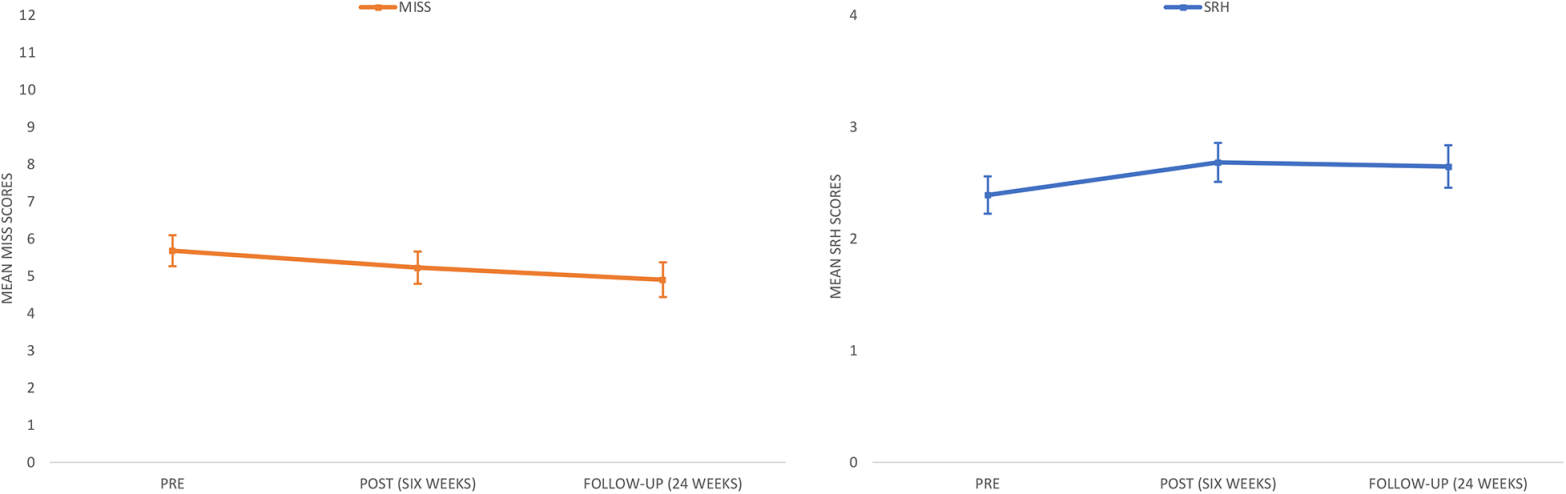
Estimated marginal means of MISS and SRH across three assessments, with 95% confidence intervals.

There was a significant baseline relationship between depression (CES-DC) and Insomnia, such that a one-point increase in depression was associated with an average 0.028-point increase in insomnia (*p* = 0.032). Overall, the tests of fixed effects showed significant improvements over time regarding symptoms of insomnia assessed with MISS [*F*(2, 176.574) = 4.165, *p* = 0.017]. Specifically, based on pairwise comparisons of the estimated marginal means from the pre-assessment, there was a non-significant average decrease on MISS of 0.455 points at post assessment (*SE* = 0.256; *p* = 0.232), and a significant average decrease of 0.779 points at the six-month follow-up (*SE* = 0.275; *p* = 0.15; Figure 1). Calculations of Cohen's d showed small effect sizes from pre- to post-assessment (*d* = 0.21) and pre- to the six-month follow-up-assessment (*d* = 0.32).

There was a significant baseline relationship between depression (CES-DC) and self-rated health, such that a one-point increase in depression was associated with an average 0.014-point decrease in self-rated health (*p* = 0.007). Overall, the tests of fixed effects showed significant improvements over time [*F*(2, 186.019) = 4.998, *p* = 0.08] on self-rated health (SRH). Specifically, based on pairwise comparisons of the estimated marginal means from the pre-assessment from the pre-assessment, there was a significant average increase of 0.292 points at post-assessment (*SE* = 0.101; *p* = 0.013), and an average increase of 0.255 points at the six-month follow-up, just at the level of significance (*SE* = 0.106; *p* = 050; Figure 1). Calculations of Cohen's *d* showed small effect sizes from pre- to post-assessment (*d* = 0.30) and pre- to the six-month follow-up-assessment (*d* = 0.25). Estimates of the fixed effects and related test statistics for the models including MISS and SRH are presented in Table 3. In Table 3, estimates of the time-invariant predictors represent the average value of the dependent variable following a one-point increase in the predictor. The estimate of the time variable, included as a factor, represents average change in the predictor from the reference time-point, Time 2 (6-month follow-up) in these analyses.

**Table 3:** The estimates of fixed effects for the models with symptoms of insomnia (MISS) and self-rated health (SRH) as the dependent variables, and the included predictors.

Variable	Parameter	Estimate	Std. Error	95% confidence interval		
					Lower	Upper
				Sig.	Bound	Bound
Symptoms of insomnia (MISS)						
Intercept^a^		0.080	1.459	0.957	−2.584	3.013
Gender (female)		0.077	0.300	0.799	−0.518	0.671
Gender (male)		0^d^	–	–	–	–
Age		−0.039	0.087	0.654	−0.216	0.137
CES DC^b^		0.028	0.013	0.032	0.002	0.053
MISS^c^		0.753	0.046	0.000	0.661	0.845
Baseline pain intensity		0.055	0.073	0.451	−0.089	0.198
Time 0		0.779	0.275	0.005	0.237	1.320
Time 1		0.324	0.277	0.244	−0.233	0.871
Time 2		0^d^	–	–	–	–
Self-rated health (SRH)						
Intercept^a^		1.439	0.585	0.016	0.272	2.606
Gender (female)		−0.036	0.111	0.750	−0.256	0.185
Gender (male)		0^d^	–	–	–	–
Age		0.033	0.035	0.347	−0.037	0.104
CES DC^b^		−0.014	0.005	0.007	−0.025	−0.004
SRH^c^		0.497	0.066	0.000	0.366	0.629
Baseline pain intensity		−0.015	0.027	0.578	−0.069	0.039
Time 0		−0.255	0.106	0.017	−0.463	−0.046
Time 1		0.037	0.109	0.733	−0.178	0.253
Time 2		0^d^	–	–	–	–

^a^ Dependent variables: symptoms of insomnia, MISS, minimal insomnia symptom scale and self-rated health; SRH, self-rated health.

^b^ Baseline levels of symptoms of depression assessed with the CES-DC, center for epidemiological studies depression scale for children.

^c^ Baseline levels of the respective outcome variables: MISS and SRH.

^d^ The parameter set to zero represents the reference category.

## 4. Discussion

Broadly, results showed small significant improvements of symptoms of insomnia at the six-month follow-up and small significant improvements in self-rated health across assessments, providing cautionary promise for an early brief person-centred intervention delivered by school nurses, including strategies to manage stress, pain and to improve sleep.

We have not identified any other studies specifically evaluating the person-centred intervention approach taken here, regarding symptoms of insomnia or self-rated health. This makes it challenging to situate our tentative findings in relation to previous research. Thus, we briefly discuss the findings in relation to cognitive behavioral therapy (CBT), an empirically supported psychological approach for children and adolescents with chronic pain (36–38). Notably though, a systematic review found that sleep was only reported as a treatment outcome in two studies evaluating CBT for adolescents with chronic pain (39). Results from one of these two trials implied a significant but small benefit from CBT on sleep quality compared to a pain education control condition (40).

The findings in the current study adds to this previous limited research, and points to the need for further research on the efficacy of person-centred school-based brief interventions, in regard to both symptoms of insomnia and self-rated health. As such HOPE may comprise an early avenue of care, provided prior to interventions in primary and tertiary care settings. We deem this a relevant approach, as availability of adequate treatment providers for children and adolescents with chronic pain and related difficulties such as insomnia, outside urban areas may be scarce and challenging to access (41). These barriers to access raises the need to develop and evaluate interventions with broad reach. In this regard, the current study is a face-to face alternative in school health, as part of a strategy aiming at broad access to early interventions for adolescents with chronic pain.

The above being said, in the current study the overall effect sizes were small, and the significant improvements on symptoms of insomnia were found at the six-month follow-up, and not at post-assessment. The small and delayed effects as illustrated by the results may be the result of a lack of, or too low a dosage of, specific interventions addressing symptoms of insomnia. In contrast to CBT-based approaches, that provide systematic directed interventions to address symptoms of insomnia, the current approach in HOPE was one of providing advice in a non-directive manner. In addition to factors more directly related to the intervention such as content and dosage, the delayed change could be due to factors outside the intervention.

These aspects highlight the need to take into consideration a number of limitations with the current study when assessing the results discussed so far. A core limitation of the study is the inability to draw firm causal conclusions as to the effect of the intervention, due to the non-randomized approach taken here. This means that results presented here could be the consequence of factors external to and independent of the intervention, such as participants receiving support from external caregivers to address symptoms of insomnia. A related, limitation has to do with the lack of control of additional control variables, given the non-randomized approach, an example being data on the number of participants receiving specific interventions addressing symptoms of insomnia outside of HOPE. Another limitation has to do with the systematic loss of participants to follow-up and the risk that the findings are a function of specific factors pertaining to that loss of data. For example, it may be the case that participants that responded poorly to the intervention did not answer the questionnaires and that results were inflated as a consequence of that.

Future studies need to identify ways to consolidate and improve on the approach taken in this study, as well as in our previously published study (15). Additionally, future intervention studies should build into their design the ability to investigate processes of change, that is for whom and under which circumstances potential changes takes place, i.e., the evaluation of treatment predictors, moderators and mediators. Avenues to explore could include the role of symptoms in relation to insomnia and self-rated-health, such as pain intensity or depression, for which we found a baseline correlation with both insomnia and self-rated health in the current study. Another approach could include investigating the role of the specific target of the intervention, self-efficacy, on for example pain related variables, such as pain intensity or pain impact. Lastly, a qualitative study exploring the adolescents’ experiences of participating in the HOPE intervention illustrated that partnership with the school nurse, was experienced as central in strengthening the adolescent's own confidence and ability to deal with chronic pain (42). This illustrates the potential role of the alliance between the adolescent and school nurse as a relevant process variable to explore further.

In conclusion, the results tentatively illustrated small to moderate significant improvements over time in regard to symptoms of insomnia and self-rated health following the HOPE intervention. Although caution is needed when assessing the findings and further study is warranted, the results illustrate the potential utility of an accessible brief intervention to address symptoms of insomnia provided in a school context as an avenue to provide an early intervention, prior to treatment in primary and tertiary care settings.

## Data Availability

The datasets presented in this article are not readily available because the General Data Protection Regulation (GDPR) applies to the data collected in this study, i.e., any information that refers to an identified or identifiable natural person. The GDPR applies in principle to every kind of operation and activity and regardless of who carries out the processing of this personal data. It thus applies to companies, associations, organisations, authorities and private individuals. Requests to access the datasets should be directed to mike.kemani@regionstockholm.se.
